# Prognostic Factors and Nomogram‐Based Prediction Models for Colorectal Cancer Patients With Synchronous Peritoneal Metastasis Undergoing Cytoreductive Surgery: A Retrospective Cohort Study

**DOI:** 10.1002/cam4.71464

**Published:** 2025-12-26

**Authors:** Xiaoxu Ge, Guanli Yang, Huiming Wu, Lei Liu, Ming Zhou, Akao Zhu, Xiangxing Kong, Jingjing Wu, Yeting Hu, Kefeng Ding, Lifeng Sun, Jian Wang

**Affiliations:** ^1^ Department of Colorectal Surgery and Oncology (Key Laboratory of Cancer Prevention and Intervention, China National Ministry of Education), The Second Affiliated Hospital Zhejiang University School of Medicine Hangzhou Zhejiang China; ^2^ Center for Medical Research and Innovation in Digestive System Tumors, Ministry of Education Zhejiang University School of Medicine Hangzhou Zhejiang China; ^3^ Department of Pathology & Pathophysiology Zhejiang University School of Medicine Hangzhou Zhejiang China

**Keywords:** colorectal cancer, cytoreductive surgery, prognostic factors, synchronous peritoneal metastasis

## Abstract

**Background:**

Colorectal cancer (CRC) with synchronous peritoneal metastasis (SPM) presents poor prognosis and complex treatment challenges. This study aimed to identify independent prognostic factors and develop nomogram‐based prediction models for overall survival (OS) and progression‐free survival (PFS) in CRC patients with SPM (CRC‐SPM) undergoing cytoreductive surgery (CRS).

**Methods:**

We retrospectively analyzed 218 CRC‐SPM treated with CRS between October 2010 and April 2022. Univariate and multivariate Cox regression analyses were used to identify independent prognostic factors for OS and PFS. Nomogram models were constructed based on these factors, and their predictive accuracy was assessed using calibration curves, ROC curves, and Decision Curve Analysis (DCA).

**Results:**

For OS, independent factors included age > 65 years (HR = 2.464, *p* = 0.005), N2 stage (HR = 2.720, *p* = 0.005), > 13 lymph nodes resected (HR = 0.496, *p* = 0.018), and postoperative chemotherapy (HR = 0.300, *p* = 0.020). For PFS, independent factors were age > 65 years (HR = 1.578, *p* = 0.040), concurrent liver metastasis (HR = 1.664, *p* = 0.016), > 14 PCI score (HR = 1.630, *p* = 0.031), and presence of ascites (HR = 1.706, *p* = 0.011). Nomogram models for predicting OS and PFS had AUC values of 0.717, 0.759, and 0.773 for OS, and 0.659, 0.689, and 0.790 for PFS at 1, 2, and 3 years, respectively.

**Conclusion:**

This study identified key prognostic factors and developed reliable nomogram models for predicting OS and PFS in CRC‐SPM. The findings highlight the importance of postoperative chemotherapy and intraoperative lymph node dissection and suggest focusing on high‐risk factors such as age and N2 stage in clinical practice. The nomogram models provide a valuable tool for personalized prognosis assessment and treatment planning.

**Abbreviations**
AUC: the area under the receiver operating characteristic curve
CC score: Completeness of Cytoreduction Score
CIs: confidence intervals
COMPASS: Colorectal Peritoneal Metastases Prognostic Surgical Score
CRC: colorectal cancer
CRC‐SPM: colorectal cancer patients with synchronous peritoneal metastasis
CRS: cytoreductive surgery
DCA: decision curve analysis
HRs: hazard ratios
KM: Kaplan–Meier
MDT: multidisciplinary team
OS: overall survival
PCI: peritoneal cancer index
PFS: progression‐free survival
PM: peritoneal metastases
PSDSS: Peritoneal Surface Disease Severity Score
ROC: receiver operating characteristic
SPM: synchronous peritoneal metastasis

## 1 Introduction

Colorectal cancer (CRC) remains a global health burden [1], with China reporting 517,100 new cases and 240,000 deaths in 2024 [2]. Peritoneal metastasis (PM) occurs in ~17% of metastatic CRC cases and represents a major cause of mortality [3]. The management of CRC with synchronous peritoneal metastasis (SPM) presents significant challenges due to the aggressive characteristics of the disease and the complexities involved in addressing both the primary tumor and metastatic lesions. The primary therapeutic modalities include cytoreductive surgery (CRS), systemic chemotherapy, targeted therapies, and emerging strategies like neoadjuvant treatment [4]. These approaches are often used in combination, depending on the individual patient's condition, to improve treatment outcomes and survival rates. CRS has been considered a crucial treatment modality for SPM, aiming to maximize tumor debulking while addressing both primary and metastatic lesions [5]. In recent years, with the advancement of research into the biological behavior and metastatic mechanisms of CRC, an increasing number of prognostic factors have been identified and applied in clinical practice. These factors include patient age [6], tumor marker levels [7], perineural invasion [8], lymph node dissection count [9], and pathological indicators [10], among others. However, the independent prognostic factors for CRC patients with SPM (CRC‐SPM) who have undergone CRS remain inadequately defined.

Over the past few years, prognosis models based on multi‐factorial analysis have attracted growing attention. By integrating multiple independent prognostic factors, these models can more accurately predict patients' survival and recurrence risks, providing stronger support for clinical decision‐making. For example, nomograms, as a visual prediction tool, have been widely used for prognostic evaluation in various cancers and have demonstrated superior predictive performance compared to traditional staging systems [11, 12, 13].

Therefore, this study aims to systematically analyze the clinical and pathological characteristics of CRC‐SPM who have undergone CRS, identify independent prognostic factors, and construct a multi‐factorial prognostic scoring model. This model seeks to provide a new foundation for the development of clinical treatment strategies and individualized prognostic evaluations for patients.

## 2 Methods

### 2.1 Ethics and Patients

This study included CRC‐SPM who underwent CRS at the Second Affiliated Hospital of Zhejiang University School of Medicine between October 2010 and April 2022. The study was approved by the Institutional Review Board of the Second Affiliated Hospital of Zhejiang University School of Medicine (Approval No.: 2025‐0826) and was conducted in accordance with the Declaration of Helsinki. All patients included in the study provided signed informed consent.

### 2.2 Inclusion and Exclusion Criteria

Patients were included in the study based on the following criteria: (1) Diagnosis of CRC‐SPM confirmed by postoperative pathological examination following CRS; (2) Underwent the first CRS at the Second Affiliated Hospital of Zhejiang University School of Medicine.

Exclusion criteria were as follows: (1) Metachronous peritoneal metastasis; (2) Clinical imaging or other diagnostic methods suggesting possible SPM, but not confirmed by postoperative pathological examination; (3) SPM treated with CRS at another institution or not undergoing CRS; (4) Presence of other primary malignant tumors.

### 2.3 Surgical Procedure and Assessment

CRS was performed aiming to maximize tumor debulking while addressing both primary and metastatic lesions. The completeness of cytoreduction was assessed using the CC score (Completeness of Cytoreduction Score), which is defined as follows: CC0 indicates no macroscopic residual tumor; CC1 indicates residual tumor ≤ 0.25 cm in maximum diameter; CC2 indicates residual tumor > 0.25 cm and ≤ 2.5 cm in maximum diameter; and CC3 indicates residual tumor > 2.5 cm or multiple residual tumors.

### 2.4 Clinical Follow‐Up

The majority of patients were followed up at the outpatient clinic of the Second Affiliated Hospital of Zhejiang University School of Medicine. During follow‐up visits, key assessments included tumor marker measurements and contrast‐enhanced CT scans to monitor for recurrence or progression of the disease. The frequency of these follow‐ups typically ranged from every 3–6 months, depending on the patient's clinical condition and the physician's recommendation. If necessary, PET‐CT scans were also performed to further assess any potential metastasis or recurrence that may not be detected by CT alone. Additionally, all patients received telephone follow‐ups every 6 months to update their health status and ensure ongoing monitoring of their condition.

### 2.5 Established Prognostic Scoring Systems

To comprehensively evaluate the performance of our nomogram model, we compared it with three established prognostic scoring systems: the Peritoneal Surface Disease Severity Score (PSDSS), the Peritoneal Cancer Index (PCI) score, and the Colorectal Peritoneal Metastases Prognostic Surgical Score (COMPASS). These scoring systems have been widely used to assess the severity of peritoneal disease and predict patient outcomes.

The PSDSS evaluates the severity of peritoneal surface disease based on preoperative clinical symptoms, the extent of peritoneal carcinomatosis, and the histopathologic features of the primary tumor [14]. It is divided into four stages, with higher stages indicating more severe disease and worse prognosis. The PCI Score quantifies the extent of PM through a visual assessment during surgery, with scores ranging from 0 to 39 [15]. Higher PCI scores are associated with more extensive peritoneal involvement and worse prognosis. The COMPASS is a prognostic nomogram that predicts survival outcomes in patients with colorectal peritoneal metastases treated with cytoreductive surgery and hyperthermic intraperitoneal chemotherapy [16]. The COMPASS score is based on age, PCI score, locoregional lymph node status, and signet ring cell histology.

### 2.6 Statistical Analysis

In the present study, overall survival (OS) was defined as the time from the date of diagnosis of CRC‐SPM to the date of death or last follow‐up. This definition captures the entire disease course from diagnosis to the final outcome, including the impact of preoperative treatments and the CRS itself. Progression‐free survival (PFS) was defined as the time from the date of CRS to the date of disease progression or last follow‐up. This definition focuses on evaluating the effectiveness of CRS and postoperative treatments in controlling disease progression. These definitions provide a comprehensive assessment of treatment outcomes from different perspectives. The cutoff values for tumor size, PCI score, and number of lymph nodes resected were determined based on the optimal cutoff values calculated for OS outcomes. Although our study assessed two primary endpoints, namely OS and PFS, the cutoff values were specifically derived from the OS data to ensure consistency and clinical relevance in our prognostic analyses. We selected a BMI cutoff of 24 and an age cutoff of 50 years based on established clinical guidelines and prior research. A BMI of 24 or higher is classified as overweight according to the World Health Organization and the Chinese Obesity Problem Working Group, which is associated with increased risks of chronic diseases and potentially higher surgical risks and worse prognoses [17].

Univariate and multivariate Cox regression analyses were conducted to identify potential prognostic factors influencing OS and PFS. The proportional hazards assumption was tested for each variable included in the Cox regression models. Variables that were statistically significant in the univariate analysis (*p* < 0.1) were subsequently included in the multivariate analysis. Stepwise selection was applied for model selection in the multivariate Cox regression analysis. Hazard ratios (HRs) with 95% confidence intervals (CIs) were calculated for each factor. All statistical analyses were performed using IBM SPSS Statistics (version 26.0, IBM Corp.). Forest plots to visualize the results of the statistically significant multivariate Cox regression analysis were generated using the “ggplot2” package in R. A *p*‐value of < 0.05 was considered statistically significant. The consistency of the effect of HIPEC on efficacy was evaluated across 36 subgroups, without adjustment for multiple comparisons.

Prognostic nomograms and calibration curves were developed using R Studio (version 4.2.1). The nomogram was constructed based on the results of the multivariate Cox regression analysis (*p* < 0.05). To assess the predictive performance of the nomogram, the area under the receiver operating characteristic curve (AUC) was calculated. The AUC curves for 1‐, 2‐, and 3‐year outcomes were plotted using the ‘timeROC’ package in R. Decision Curve Analysis (DCA) was performed to assess the clinical utility of the nomogram models at 1, 2, and 3 years. Net benefits at various threshold probabilities were calculated using the ‘dca’ function in R, comparing the nomogram models to alternative strategies. Kaplan–Meier (KM) survival curves were generated using the ‘survival’ and ‘survminer’ packages in R.

## 3 Results

### 3.1 Patient Characteristics

**FIGURE 1:**
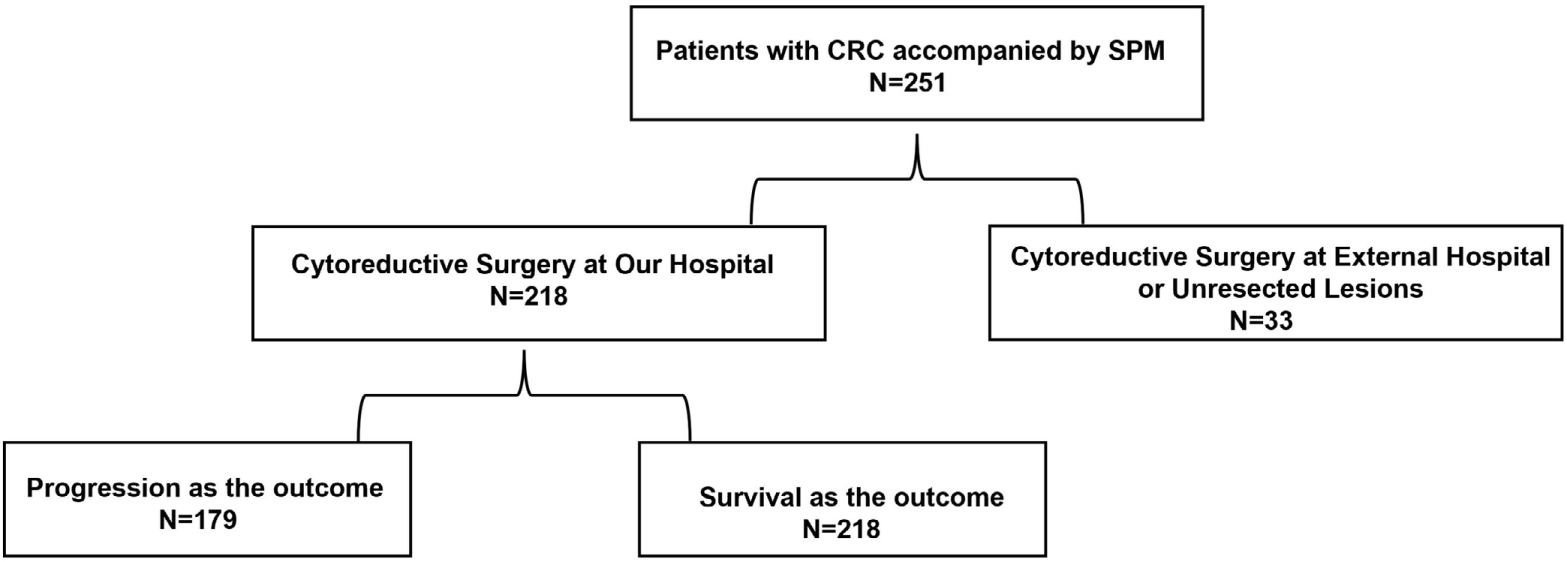
Flowchart of the experimental cohort design.

Between October 2010 and April 2022, a total of 251 CRC‐SPM were treated at our hospital (Figure 1). Among them, 218 patients underwent their primary CRS at our hospital, and of these, 179 patients had available follow‐up data regarding recurrence, while all 218 patients had survival outcome data (see Table 1). A total of 218 CRC‐SPM patients were included in our study, of whom seven were dMMR patients. Notably, only three of these seven dMMR patients received immunotherapy. This is due to the study period. In China, immunotherapy was not incorporated into the treatment recommendations for CRC until the 2019 CSCO guidelines [18]. Consequently, the remaining four dMMR patients did not undergo immunotherapy due to the prevailing treatment protocols at the time. Additionally, among the 19 patients who received immunotherapy, 16 were pMMR patients, all enrolled in clinical trials. Specifically, 15 pMMR patients received a combination of immunotherapy, chemotherapy, and targeted therapy, while one received a combination of immunotherapy and targeted therapy.

**TABLE 1:** Baseline characteristics of 218 patients.

Characteristics	OS outcomes	PFS outcomes
Total patients number, *n*	218	179
Year of CRS performed, *n* (%)		
2010–2016	48 (22%)	34 (19%)
2017–2022	170 (78%)	145 (81%)
Gender, *n* (%)		
Female	99 (45.4%)	79 (44.1%)
Male	119 (54.6%)	100 (55.9%)
Age, *n* (%)		
≤ 65	136 (62.4%)	119 (66.5%)
> 65	82 (37.6%)	60 (33.5%)
BMI, *n* (%)		
< 24	152 (69.7%)	124 (69.3%)
≥ 24	62 (28.4%)	53 (29.6%)
Unknown	4 (1.8%)	2 (1.1%)
Smoking history, *n* (%)		
No	158 (72.5%)	126 (70.4%)
Yes	60 (27.5%)	53 (29.6%)
Alcohol history, *n* (%)		
No	164 (75.2%)	132 (73.7%)
Yes	54 (24.8%)	47 (26.3%)
Hypertension history, *n* (%)		
No	156 (71.6%)	129 (72.1%)
Yes	62 (28.4%)	50 (27.9%)
Diabetes history, *n* (%)		
No	187 (85.8%)	158 (88.3%)
Yes	31 (14.2%)	21 (11.7%)
Liver metastasis, *n* (%)		
No	136 (62.4%)	116 (64.8%)
Yes	82 (37.6%)	63 (35.2%)
Preoperative CEA, *n* (%)		
≤ 5 ng/mL	62 (28.4%)	55 (30.7%)
> 5 ng/mL	135 (61.9%)	109 (60.9%)
Unknown	21 (9.6%)	15 (8.4%)
Preoperative CA199, *n* (%)		
≤ 37 U/mL	92 (42.2%)	80 (44.7%)
> 37 U/mL	102 (46.8%)	82 (45.8%)
Unknown	24 (11%)	17 (9.5%)
Preoperative CA125, *n* (%)		
≤ 35 U/mL	105 (48.2%)	89 (49.7%)
> 35 U/mL	87 (39.9%)	71 (39.7%)
Unknown	26 (11.9%)	19 (10.6%)
Preoperative CA242, *n* (%)		
≤ 20 U/mL	95 (43.6%)	82 (45.8%)
> 20 U/mL	91 (41.7%)	72 (40.2%)
Unknown	32 (14.7%)	25 (14%)
Preoperative chemotherapy, *n* (%)		
No	178 (81.7%)	146 (81.6%)
Yes	40 (18.3%)	33 (18.4%)
Preoperative radiotherapy, *n* (%)		
No	216 (99.1%)	177 (98.9%)
Yes	2 (0.9%)	2 (1.1%)
Preoperative immunotherapy, *n* (%)		
No	218 (100%)	179 (100%)
Yes	0 (0%)	0 (0%)
Preoperative targeted therapy, *n* (%)		
No	195 (89.4%)	161 (89.9%)
Yes	23 (10.6%)	18 (10.1%)
CRS type, *n* (%)		
Open surgery	141 (64.7%)	113 (63.1%)
Laparoscopic surgery	77 (35.3%)	66 (36.9%)
Acute abdominal symptoms, *n* (%)		
No	153 (70.2%)	130 (72.6%)
Yes	65 (29.8%)	49 (27.4%)
BRAF mutation status, *n* (%)^a^		
BRAF wild‐type	166 (76.1%)	133 (74.3%)
BRAF V600E mutation	13 (6%)	12 (6.7%)
Unknown	39 (17.9%)	34 (19%)
Microsatellite status, *n* (%)		
MSS	32 (14.7%)	29 (16.2%)
MSI	1 (0.5%)	1 (0.6%)
Unknown	185 (84.9%)	149 (83.2%)
Mismatch repair gene status, *n* (%)		
pMMR	184 (84.4%)	156 (87.2%)
dMMR	7 (3.2%)	7 (3.9%)
Unknown	27 (12.4%)	16 (8.9%)
T stage, *n* (%)		
T2‐T3	51 (23.4%)	45 (25.1%)
T4	164 (75.2%)	133 (74.3%)
Unknown	3 (1.4%)	1 (0.6%)
Tumor margin status of primary lesion, *n* (%)		
R0	207 (95%)	174 (97.2%)
R1‐R2	7 (3.2%)	4 (2.2%)
Unknown	4 (1.8%)	1 (0.6%)
Tumor size, *n* (%)		
≤ 3.5 cm	57 (26.1%)	50 (27.9%)
> 3.5 cm	149 (68.3%)	123 (68.7%)
Unknown	12 (5.5%)	6 (3.4%)
Neural invasion in primary tumor, *n* (%)		
No	59 (27.1%)	51 (28.5%)
Yes	134 (61.5%)	114 (63.7%)
Unknown	25 (11.5%)	14 (7.8%)
Vascular invasion in primary tumor, *n* (%)		
No	77 (35.3%)	65 (36.3%)
Yes	122 (56%)	104 (58.1%)
Unknown	19 (8.7%)	10 (5.6%)
N stage, *n* (%)		
N0‐N1	120 (55%)	96 (53.6%)
N2	90 (41.3%)	79 (44.1%)
Unknown	8 (3.7%)	4 (2.2%)
Number of lymph nodes resected, *n* (%)		
≤ 13	83 (38.1%)	63 (35.2%)
> 13	125 (57.3%)	111 (62%)
Unknown	10 (4.6%)	5 (2.8%)
Location of primary tumor, *n* (%)		
Right‐sided colon	111 (50.9%)	90 (50.3%)
Left‐sided colon	107 (49.1%)	89 (49.7%)
Pathological type, *n* (%)		
Adenocarcinoma	166 (76.1%)	139 (77.7%)
Mucinous adenocarcinoma	42 (19.3%)	31 (17.3%)
Signet ring cell carcinoma	10 (4.6%)	9 (5%)
Pathological differentiation degree, *n* (%)		
Well—moderately differentiated	116 (53.2%)	96 (53.6%)
Poorly differentiated	64 (29.4%)	56 (31.3%)
Unknown	38 (17.4%)	27 (15.1%)
Macroscopic type, *n* (%)		
Infiltrative	1 (0.5%)	1 (0.6%)
Ulcerative	136 (62.4%)	118 (65.9%)
Protruding	51 (23.4%)	40 (22.3%)
Unknown	30 (13.8%)	20 (11.2%)
CC score, *n* (%)		
CC0	172 (78.9%)	144 (80.4%)
CC1‐CC3	46 (21.1%)	35 (19.6%)
PCI score, *n* (%)		
≤ 14	153 (70.2%)	129 (72.1%)
> 14	65 (29.8%)	50 (27.9%)
Ascites, *n* (%)		
No	105 (48.2%)	91 (50.8%)
Yes	113 (51.8%)	88 (49.2%)
Invasion of small intestine, *n* (%)		
No	170 (78%)	145 (81%)
Yes	48 (22%)	34 (19%)
Intraoperative blood loss, *n* (%)		
< 100 mL	89 (40.8%)	76 (42.5%)
≥ 100 mL	127 (58.3%)	102 (57%)
Unknown	2 (0.9%)	1 (0.6%)
HIPEC performed, *n* (%)		
No	145 (66.5%)	115 (64.2%)
Yes	73 (33.5%)	64 (35.8%)
Postoperative complications, *n* (%)		
No	200 (91.7%)	165 (92.2%)
Yes	18 (8.3%)	14 (7.8%)
Average postoperative hospital stay, *n* (%)		
< 10 days	115 (52.8%)	96 (53.6%)
≥ 10 days	103 (47.2%)	83 (46.4%)
Postoperative chemotherapy, *n* (%)		
No	19 (8.7%)	18 (10.1%)
Yes	169 (77.5%)	156 (87.2%)
Unknown	30 (13.8%)	5 (2.8%)
Postoperative radiotherapy, *n* (%)		
No	178 (81.7%)	164 (91.6%)
Yes	10 (4.6%)	10 (5.6%)
Unknown	30 (13.8%)	5 (2.8%)
Postoperative immunotherapy, *n* (%)		
No	168 (77.1%)	154 (86%)
Yes	19 (8.7%)	19 (10.6%)
Unknown	31 (14.2%)	6 (3.4%)
Postoperative targeted therapy, *n* (%)		
No	70 (32.1%)	58 (32.4%)
Yes	117 (53.7%)	115 (64.2%)
Unknown	31 (14.2%)	6 (3.4%)
Progression, *n* (%)		
No	31 (14.2%)	
Yes	148 (67.9%)	
Unknown	39 (17.9%)	
Number of progression sites, *n* (%)		
1	110 (50.5%)	
> 1	38 (17.4%)	
No Progression/Unknown	70 (32.1%)	

^a^ BRAF wild‐type/V600E mutation status was derived from immunohistochemical pathology reports.

**FIGURE 2:**
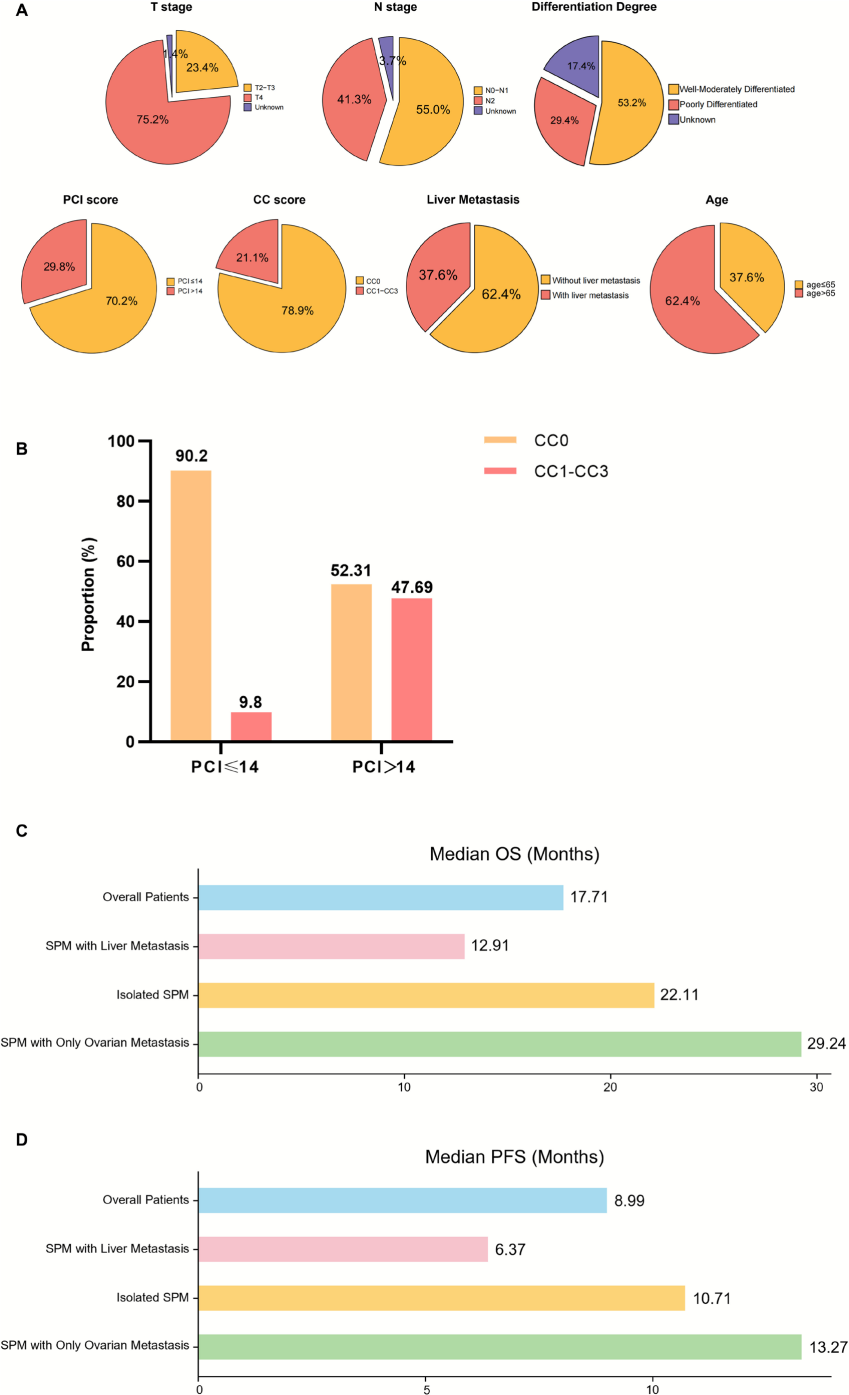
Distribution of clinical characteristics. (A) Distribution of selected clinical characteristics in the patient cohort. (B) Postoperative CRS CC Scores in patients with different PCI scores. (C, D) Bar charts illustrating the median OS (C) and PFS (D) durations.

Of the 218 patients, 37.6% were over 65 years of age, while 62.4% were 65 years old or younger. Following CRS, 78.9% had a postoperative CC score of CC0, and 21.1% had a CC score of CC1‐CC3. The extent of peritoneal involvement, as measured by the PCI, was ≤ 14 in 70.2% of patients and > 14 in 29.8%. Postoperative pathological analysis revealed that 55% of patients had N0‐N1 staging, while 41.3% had N2. Regarding the T staging, 23.4% were T2–T3, and 75.2% were T4. In terms of pathological differentiation degree, 53.2% were classified as well‐moderately differentiated, and 29.4% as poorly differentiated. Among the 218 patients with SPM, 37.6% also had concurrent liver metastasis (Figure 2A). Additionally, the distribution of postoperative CC scores varied across different PCI groups: in patients with a PCI ≤ 14, 90.2% had a CC score of CC0, while 9.8% had a CC score of CC1‐CC3; in patients with a PCI > 14, 52.31% had a CC score of CC0, and 47.69% had a CC score of CC1‐CC3 (Figure 2B). This suggests that a higher degree of peritoneal involvement is associated with increased surgical complexity.

In our study, the median progression‐free survival (PFS) was 8.99 months (95% CI: 7.88–11.33 months), with 1‐, 2‐, 3‐, and 5‐year PFS rates of 33.52%, 13.97%, 6.15%, and 1.12%, respectively. The median overall survival (OS) was 17.71 months (95% CI: 14.82–22.11 months), with 1‐, 2‐, 3‐, and 5‐ OS rates of 64.22%, 37.16%, 20.18%, and 5.50%, respectively. Additionally, the median overall survival was 23.95 months (95% CI: 16.72–31.60 months) in patients without liver metastasis, compared with 12.91 months (95% CI: 10.51–17.74 months) in those with liver metastasis. The median PFS was 10.71 months (95% CI: 8.90–13.27 months) in patients without liver metastasis and 6.44 months (95% CI: 4.17–9.82 months) in those with liver metastasis. In patients with isolated PM (without any extra‐peritoneal metastasis), the median OS was 22.11 months (95% CI: 16.56–36.24 months) and the median PFS was 10.71 months (95% CI: 8.77–12.98 months). For patients with isolated PM and concurrent ovarian metastasis only, the median OS was 29.24 months (95% CI: 25–49.47 months) and the median PFS was 13.27 months (95% CI: 9.10–24.64 months) (Figure 2C,D).

### 3.2 Independent Prognostic Factors

This section aims to analyze the independent factors influencing survival and recurrence in patients with CRC accompanied by SPM. Univariate and multivariate Cox regression analyses were performed on the clinical characteristics of the patients. Additionally, since none of the patients received preoperative immunotherapy and due to significant missing data on microsatellite status, we excluded these two factors from our subsequent analysis (see Table 1).

In the univariate analysis of OS, several factors showed statistically significant differences (*p* < 0.1), including: age > 65 years (HR = 1.817, 95% CI: 1.335–2.472, *p* < 0.001), history of hypertension (HR = 1.385, 95% CI: 1.001–1.916, *p* = 0.049), history of diabetes (HR = 1.926, 95% CI: 1.272–2.915, *p* = 0.002), liver metastasis (HR = 1.603, 95% CI: 1.173–2.190, *p* = 0.003), preoperative CA199 > 37 U/mL (HR = 1.550, 95% CI: 1.122–2.142, *p* = 0.008), preoperative CA125 > 35 U/mL (HR = 1.473, 95% CI: 1.066–2.036, *p* = 0.019), preoperative CA242 > 20 U/mL (HR = 1.573, 95% CI: 1.131–2.188, *p* = 0.007), BRAF mutation status (HR = 1.964, 95% CI: 1.056–3.655, *p* = 0.033), tumor size > 3.5 cm (HR = 1.613, 95% CI: 1.124–2.316, *p* = 0.010), vascular invasion (HR = 2.164, 95% CI: 1.542–3.038, *p* < 0.001), N stage of N2 (HR = 1.812, 95% CI: 1.322–2.485, *p* < 0.001), lymph node dissection of > 13 nodes (HR = 0.692, 95% CI: 0.506–0.946, *p* = 0.021), poorly differentiated (HR = 1.402, 95% CI: 0.986–1.994, *p* = 0.060), CC score of CC1‐CC3 (HR = 1.417, 95% CI: 0.995–2.017, *p* = 0.053), PCI score > 14 (HR = 1.854, 95% CI: 1.346–2.553, *p* < 0.001), presence of ascites (HR = 1.504, 95% CI: 1.108–2.041, *p* = 0.009), HIPEC performed (HR = 0.541, 95% CI: 0.388–0.756, *p* < 0.001), postoperative complications (HR = 1.654, 95% CI: 1.001–2.734, *p* = 0.050), postoperative chemotherapy (HR = 0.493, 95% CI: 0.296–0.823, *p* = 0.007), postoperative radiotherapy (HR = 0.493, 95% CI: 0.217–1.123, *p* = 0.092), postoperative immunotherapy (HR = 0.588, 95% CI: 0.325–1.065, *p* = 0.080), and postoperative targeted therapy (HR = 0.601, 95% CI: 0.429–0.843, *p* = 0.003).

**FIGURE 3:**
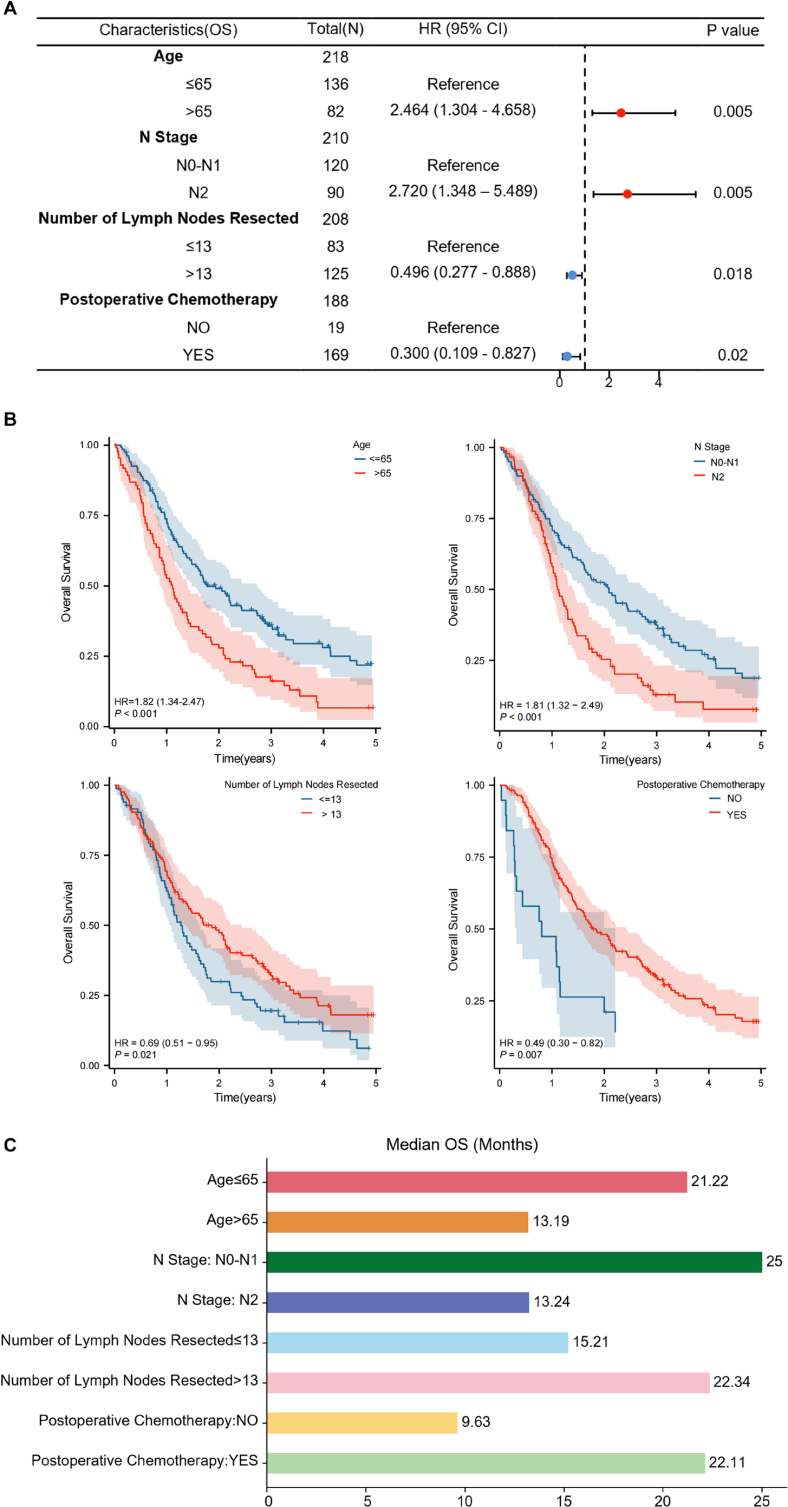
Multivariate cox regression analysis for prognostic factors of overall survival. (A) The forest plot displays hazard ratios (HR) with 95% confidence intervals (CI) and *p* values for each independent prognostic factor associated with overall survival (OS). Red dots denote unfavorable prognostic factors, while blue dots indicate favorable prognostic factors. The vertical dashed line represents the null effect (HR = 1), and factors with CIs not crossing this line are considered statistically significant. (B) The Kaplan–Meier curve shows factors significantly affecting OS in the multivariate Cox regression analysis. (C) Bar charts separately illustrate the median survival times of independent prognostic factors for OS.

In the multivariate Cox regression analysis for OS, we identified several independent prognostic factors that were statistically significant (*p* < 0.05). Specifically, age > 65 years was associated with a significantly higher risk of death compared to patients ≤ 65 years (HR = 2.464, 95% CI: 1.304–4.658, *p* = 0.005). Similarly, N2 stage, compared to N0‐N1, also showed a significantly higher risk of mortality (HR = 2.720, 95% CI: 1.348–5.489, *p* = 0.005). On the other hand, patients with more than 13 lymph nodes dissected had a significantly reduced risk of death (HR = 0.496, 95% CI: 0.277–0.888, *p* = 0.018). Lastly, receiving postoperative chemotherapy was associated with a significantly lower risk of death (HR = 0.300, 95% CI: 0.109–0.827, *p* = 0.020) (see Table 2 and Figure 3).

**TABLE 2:** Univariate and multivariate cox regression analysis for overall survival outcomes.

Characteristics	Total (*N*)	Univariate analysis		Multivariate analysis	
		Hazard ratio (95% CI)	*p*	Hazard ratio (95% CI)	*p*
Year of CRS performed	218				
2010–2016	48	Reference			
2017–2022	170	0.847 (0.598–1.199)	0.348		
Gender	218				
Female	99	Reference			
Male	119	1.018 (0.751–1.379)	0.909		
Age	218				
≤ 65	136	Reference		Reference	
> 65	82	1.817 (1.335–2.472)	**< 0.001**	2.464 (1.304–4.658)	**0.005**
BMI	214				
< 24	152	Reference			
≥ 24	62	0.819 (0.581–1.154)	0.253		
Smoking history	218				
No	158	Reference			
Yes	60	0.993 (0.709–1.392)	0.969		
Alcohol history	218				
No	164	Reference			
Yes	54	0.956 (0.674–1.356)	0.799		
Hypertension history	218				
No	156	Reference		Reference	
Yes	62	1.385 (1.001–1.916)	**0.049**	1.171 (0.557–2.462)	0.678
Diabetes history	218				
No	187	Reference		Reference	
Yes	31	1.926 (1.272–2.915)	**0.002**	1.162 (0.502–2.693)	0.726
Liver metastasis	218				
No	136	Reference		Reference	
Yes	82	1.603 (1.173–2.190)	**0.003**	1.661 (0.868–3.178)	0.125
Preoperative CEA	197				
≤ 5 ng/mL	62	Reference			
> 5 ng/mL	135	1.115 (0.790–1.574)	0.536		
Preoperative CA199	194				
≤ 37 U/mL	92	Reference		Reference	
> 37 U/mL	102	1.550 (1.122–2.142)	**0.008**	1.403 (0.606–3.244)	0.429
Preoperative CA125	192				
≤ 35 U/mL	105	Reference		Reference	
> 35 U/mL	87	1.473 (1.066–2.036)	**0.019**	1.146 (0.585–2.246)	0.690
Preoperative CA242	186				
≤ 20 U/mL	95	Reference		Reference	
> 20 U/mL	91	1.573 (1.131–2.188)	**0.007**	1.103 (0.446–2.729)	0.832
Preoperative chemotherapy	218				
No	178	Reference			
Yes	40	0.886 (0.597–1.316)	0.550		
Preoperative radiotherapy	218				
No	216	Reference			
Yes	2	1.026 (0.254–4.145)	0.971		
Preoperative targeted therapy	218				
No	195	Reference			
Yes	23	0.929 (0.576–1.498)	0.763		
CRS type	218				
Open surgery	141	Reference			
Laparoscopic surgery	77	0.866 (0.629–1.192)	0.376		
Acute abdominal symptoms	218				
No	153	Reference			
Yes	65	1.098 (0.789–1.529)	0.580		
BRAF mutation status	179				
BRAF wild‐type	166	Reference		Reference	
BRAF V600E mutation	13	1.964 (1.056–3.655)	**0.033**	0.713 (0.256–1.986)	0.517
Mismatch repair gene status	191				
pMMR	184	Reference			
dMMR	7	1.105 (0.450–2.711)	0.827		
T stage	215				
T2‐T3	51	Reference			
T4	164	1.027 (0.717–1.471)	0.885		
Tumor margin status of primary lesion	214				
R0	207	Reference			
R1‐R2	7	1.632 (0.762–3.499)	0.208		
Tumor size	206				
≤ 3.5 cm	57	Reference		Reference	
> 3.5 cm	149	1.613 (1.124–2.316)	**0.010**	0.996 (0.439–2.257)	0.992
Neural invasion in primary tumor	193				
No	59	Reference			
Yes	134	1.186 (0.837–1.680)	0.338		
Vascular invasion in primary tumor	199				
No	77	Reference		Reference	
Yes	122	2.164 (1.542–3.038)	**< 0.001**	1.419 (0.660–3.050)	0.370
N stage	210				
N0‐N1	120	Reference		Reference	
N2	90	1.812 (1.322–2.485)	**< 0.001**	2.720 (1.348–5.489)	**0.005**
Number of lymph nodes resected	208				
≤ 13	83	Reference		Reference	
> 13	125	0.692 (0.506–0.946)	**0.021**	0.496 (0.277–0.888)	**0.018**
Location of primary tumor	218				
Right‐sided colon	111	Reference			
Left‐sided colon	107	0.963 (0.711–1.305)	0.808		
Pathological type^a^	218				
Adenocarcinoma	166	Reference			
Mucinous adenocarcinoma	52	1.103 (0.777–1.566)	0.583		
Pathological differentiation degree	180				
Well—moderately differentiated	116	Reference		Reference	
Poorly differentiated	64	1.402 (0.986–1.994)	**0.060**	0.860 (0.421–1.756)	0.679
Macroscopic type	188				
Infiltrative	1	Reference			
Ulcerative	136	1.098 (0.153–7.886)	0.926		
Protruding	51	1.132 (0.155–8.253)	0.902		
CC score	218				
CC0	172	Reference		Reference	
CC1‐CC3	46	1.417 (0.995–2.017)	**0.053**	1.548 (0.727–3.295)	0.257
PCI score	218				
≤ 14	153	Reference		Reference	
> 14	65	1.854 (1.346–2.553)	**< 0.001**	1.938 (0.915–4.108)	0.084
Ascites	218				
No	105	Reference		Reference	
Yes	113	1.504 (1.108–2.041)	**0.009**	1.618 (0.853–3.067)	0.141
Invasion of small intestine	218				
No	170	Reference			
Yes	48	1.313 (0.919–1.874)	0.134		
Intraoperative blood loss	216				
< 100 mL	89	Reference			
≥ 100 mL	127	1.058 (0.775–1.443)	0.723		
HIPEC performed	218				
No	145	Reference		Reference	
Yes	73	0.541 (0.388–0.756)	**< 0.001**	0.780 (0.382–1.594)	0.496
Postoperative Complications	218				
No	200	Reference		Reference	
Yes	18	1.654 (1.001–2.734)	**0.050**	0.826 (0.301–2.265)	0.710
Average postoperative hospital stay	218				
< 10 days	115	Reference			
≥ 10 days	103	0.998 (0.736–1.353)	0.989		
Postoperative chemotherapy	188				
No	19	Reference		Reference	
Yes	169	0.493 (0.296–0.823)	**0.007**	0.300 (0.109–0.827)	**0.020**
Postoperative radiotherapy	188				
No	178	Reference		Reference	
Yes	10	0.493 (0.217–1.123)	**0.092**	1.789 (0.437–7.332)	0.419
Postoperative immunotherapy	187				
No	168	Reference		Reference	
Yes	19	0.588 (0.325–1.065)	**0.080**	0.555 (0.224–1.376)	0.204
Postoperative targeted therapy	187				
No	70	Reference		Reference	
Yes	117	0.601 (0.429–0.843)	**0.003**	0.829 (0.414–1.662)	0.597
Progression	179				
No	31	Reference			
Yes	148	1.004 (0.617–1.635)	0.986		
Number of progression sites	148				
1	110	Reference			
> 1	38	1.066 (0.702–1.619)	0.764		

*Note:* In univariate analysis, boldface indicates variables with *p* < 0.1, which were selected for subsequent multivariate Cox regression. In multivariate analysis, boldface denotes independent prognostic factors.

^a^ Mucinous adenocarcinoma category includes mucinous adenocarcinoma and signet ring cell carcinoma in the dichotomous classification.

In the univariate Cox regression analysis for PFS, several factors demonstrated statistically significant differences (*p* < 0.1), including age > 65 years (HR = 1.363, 95% CI: 0.966–1.924, *p* = 0.078), history of diabetes (HR = 1.567, 95% CI: 0.964–2.549, *p* = 0.070), liver metastasis (HR = 1.498, 95% CI: 1.067–2.101, *p* = 0.019), BRAF mutation status (HR = 1.834, 95% CI: 0.954–3.525, *p* = 0.069), tumor size > 3.5 cm (HR = 1.384, 95% CI: 0.963–1.989, *p* = 0.079), vascular invasion (HR = 1.341, 95% CI: 0.952–1.889, *p* = 0.094), N2 stage (HR = 1.373, 95% CI: 0.981–1.920, *p* = 0.064), lymph node dissection of > 13 nodes (HR = 0.732, 95% CI: 0.519–1.031, *p* = 0.074), PCI score > 14 (HR = 1.697, 95% CI: 1.183–2.432, *p* = 0.004), and presence of ascites (HR = 1.550, 95% CI: 1.117–2.152, *p* = 0.009). In the multivariate analysis for PFS, age > 65 years, liver metastasis, PCI score > 14, and the presence of ascites were identified as independent prognostic factors. Specifically, patients aged > 65 years had a significantly higher risk of progression (HR = 1.578, 95% CI: 1.022–2.438, *p* = 0.040). Moreover, liver metastasis was associated with a significantly higher risk of progression (HR = 1.664, 95% CI: 1.099–2.520, *p* = 0.016). A PCI score > 14 was also associated with an increased risk of progression (HR = 1.630, 95% CI: 1.046–2.541, *p* = 0.031). The presence of ascites was associated with a higher risk of progression (HR = 1.706, 95% CI: 1.130–2.577, *p* = 0.011) (see Table S1 and Figure S1).

Additionally, although the use of HIPEC was not an independent prognostic factor for either OS or PFS in multivariate analysis, the univariate analysis of OS showed that the HR for HIPEC was 0.541 (95% CI: 0.388–0.756, *p* < 0.001). Furthermore, the potential benefit of HIPEC in patients with CRC and PM remains controversial [19]. Therefore, we further analyzed whether the application of HIPEC was effective in 36 subgroups, using OS as the outcome and the presence or absence of HIPEC as the exposure factor (see Table S2). Subgroup analysis results indicated that CRS combined with HIPEC showed better OS prognosis compared to CRS alone in both the overall population of CRC‐SPM and in 34 subgroups. However, in subgroups with different tumor sizes (≤ 3.5 cm vs. > 3.5 cm, interaction *p* = 0.033) and different mismatch repair gene status (pMMR vs. dMMR, interaction *p* = 0.042), the treatment effect of CRS combined with HIPEC showed heterogeneity. Nevertheless, since the HR values were all < 1, this still suggests that CRS combined with HIPEC provides superior efficacy.

### 3.3 Nomogram Models for Predicting OS and PFS

**FIGURE 4:**
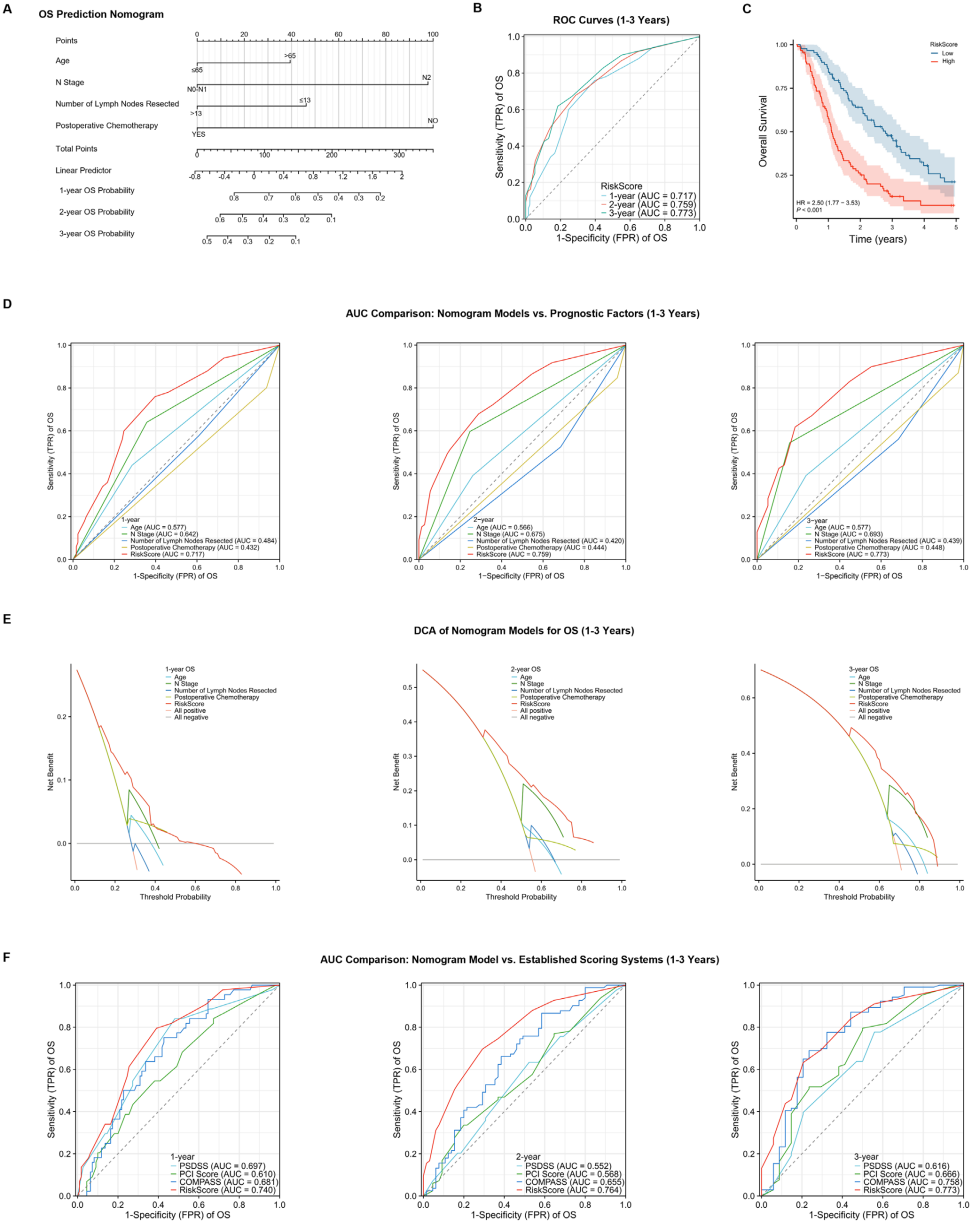
Predictive performance of nomogram models for OS. (A) Visual representation of the nomogram for OS prediction. (B) ROC curves and corresponding AUC values for OS models at 1, 2, and 3 years. (C) Kaplan–Meier survival curves stratified by low‐risk and high‐risk groups based on the nomogram models. (D) Comparison of AUC values for the nomogram models versus other independent prognostic factors at 1, 2, and 3 years. (E) DCA curves illustrating the net benefit of nomogram models for OS at varying threshold probabilities across 1–3 years. (F) Superiority of the nomogram model in predicting OS compared to established scoring systems over 1–3 years.

This section further incorporates independent prognostic factors influencing OS and PFS into nomogram models, creating separate scoring systems for OS (Figure 4A) and PFS (Figure S2A). The calibration curve (Figure S3) was used to assess the predictive accuracy of these models. The results showed that the predictions of the models closely aligned with the actual observed outcomes, indicating good calibration performance.

Figure 4B and Figure S2B present the area under the curve (AUC) values of the two models for the receiver operating characteristic (ROC) curves at 1, 2, and 3 years. The AUC values were 0.717, 0.759, and 0.773 for the OS model, and 0.659, 0.689, and 0.790 for the PFS model, respectively, demonstrating that both models have high discriminative ability.

To further validate the predictive performance of the models, Figure 4D and Figure S2D compare the predictive scores of the two nomogram models with other independent prognostic factors based on their AUC values at 1, 2, and 3 years. The results showed that the AUC values of the nomogram models (RiskScore) were higher than those of any single prognostic factor. Figure 4E and Figure S2E illustrate the Decision Curve Analysis (DCA) for both OS and PFS at 1, 2, and 3 years. The curves show that the nomogram models consistently offer a higher net benefit across various threshold probabilities compared to the scenarios of no treatment or treatment for all patients. Furthermore, we performed an extensive assessment of our nomogram models in comparison with established scoring systems—including PCI, PSDSS, and COMPASS—by analyzing AUC values derived from ROC curves for OS (as shown in Figure 4F) and PFS (as shown in Figure S2F) across a 1‐ to 3‐year period. Our findings consistently indicate that our nomogram model outperforms its counterparts. These results underscore the robustness and clinical utility of our nomogram models in forecasting prognosis outcomes, underscoring their potential as invaluable tools in the realm of personalized medicine for CRC patients afflicted with SPM.

Finally, Figure 4C and Figure S2C divide the patients into low‐risk and high‐risk groups based on the median cut‐off values of the two nomogram models and present Kaplan–Meier survival curves. The results revealed significant differences between the low‐risk and high‐risk groups for both OS and PFS (OS: HR = 2.50, *p* < 0.001; PFS: HR = 1.87, *p* < 0.001), further validating the reliability of the nomogram models. The median OS for the low‐risk group was 33.44 months (95% CI: 25.72–41.03 months), while the median OS for the high‐risk group was 13.24 months (95% CI: 11.63–16.62 months). The median PFS for the low‐risk group was 12.02 months (95% CI: 9.00–17.38 months), and the median PFS for the high‐risk group was 7.10 months (95% CI: 5.65–9.53 months).

In conclusion, the nomogram models established in this study demonstrate high accuracy and reliability in predicting OS and PFS in CRC‐SPM, offering a powerful tool for personalized clinical decision‐making.

## 4 Discussion

In our institution, the treatment strategy for CRC‐SPM is meticulously tailored to each patient through a multidisciplinary team (MDT) approach. The decision to perform CRS and integrate additional therapies such as HIPEC, systemic chemotherapy, targeted therapy, or immunotherapy is based on a comprehensive evaluation of several critical factors. These include the technical feasibility of tumor resection, the biological behavior of the tumor (e.g., histological features and molecular markers), the patient's overall health status (e.g., cardiopulmonary function and comorbidities), and the patient's treatment preferences and goals. The MDT, comprising surgical oncologists, medical oncologists, radiologists, and pathologists, carefully assesses each case to determine the optimal combination of therapies. This collaborative approach ensures that the decision to incorporate these therapies is based on a thorough assessment of the potential benefits and risks, considering the specific characteristics of the tumor and the patient's overall condition. In our study, the median OS for CRC‐SPM patients who underwent CRS was 17.71 months. For comparison, a study from the Netherlands [20] reported that the median OS for CRC‐SPM patients treated with palliative care alone was 10.0 months. Another study from China [21] indicated that the median OS for CRC‐SPM patients who did not undergo CRS was 6.0 months. These findings highlight the significant survival benefit of CRS in the management of CRC‐SPM patients.

This study specifically focuses on the prognostic factors of CRC‐SPM patients who underwent CRS. It identified key prognostic factors affecting OS and PFS and constructed a multi‐factorial prognostic scoring model based on these factors. The findings showed that age (> 65 years) and N stage (N2) were independent risk factors for OS in CRC‐SPM patients, while the number of lymph nodes resected (> 13) and postoperative chemotherapy were favorable factors for OS. Regarding PFS, age (> 65 years), liver metastasis, PCI Score (> 14), and presence of ascites were independent risk factors. These findings suggest that for elderly patients or those with N2 stage disease who have undergone CRS, an aggressive management approach is warranted. This includes intensified follow‐up and adjuvant chemotherapy to reduce mortality. Our study also underscores the importance of resecting > 13 lymph nodes during CRS, which has been shown to improve survival outcomes. This highlights the need for optimized surgical and postoperative strategies to ensure thorough lymph node dissection and effective adjuvant treatment in CRC‐SPM patients who have undergone CRS.

Furthermore, the multi‐factorial prognostic scoring model developed in this study not only demonstrated high accuracy and discriminative ability, as evidenced by calibration curves, ROC curve analysis with AUC values, and DCA, but also outperformed existing scoring models in predicting outcomes, thereby highlighting its superior predictive performance for patient prognosis. This model can assist clinicians in more accurately assessing postoperative prognosis, providing more personalized treatment recommendations for patients, and improving survival quality and treatment adherence.

Despite the achievements of this study, several limitations remain. First, the study is a single‐center retrospective analysis, which may be subject to selection bias and information bias. Second, the study did not incorporate emerging potential prognostic factors, such as differentially expressed genes. With the development of molecular diagnostic technologies, an increasing number of genetic markers have been identified as being associated with prognosis [22, 23]. Additionally, it is recognized that inflammation plays a pivotal role in the progression and metastasis of CRC. Recent studies have indicated that inflammation‐related markers could serve as prognostic factors, assessing patients' survival and recurrence risks [24, 25]. However, due to the dynamic nature of these markers throughout treatment, their levels may be influenced by various factors, making it challenging to standardize a single time point for quantification, potentially affecting the accuracy and comparability of results. Therefore, in this study, we have not included inflammation‐related indicators. Future studies could further explore the role of these emerging molecular markers and inflammation‐related markers in the prognosis of CRC‐SPM. Moreover, if feasible, external validation from multi‐center data should be included to confirm the applicability of our model, ensuring its generalizability and reliability across different populations.

Future research should indeed incorporate multi‐center prospective studies to validate our findings' generalizability and to explore new therapeutic targets along with personalized treatment strategies. Additionally, considering the significant role of inflammation in CRC, future studies should also evaluate inflammation‐related markers. A comprehensive analysis that integrates both molecular biomarkers and clinical‐pathological features, including these inflammation indicators, is likely to further refine the accuracy of prognosis assessments and treatment precision. Through multidisciplinary collaboration and the application of big data analysis, more optimized treatment strategies can be developed for CRC‐SPM, ultimately aiming to improve long‐term survival rates and quality of life.

## Author Contributions

Jian Wang, Lifeng Sun, and Kefeng Ding designed the study and supervised the research. Xiaoxu Ge, Guanli Yang, and Huiming Wu were responsible for data collection and analysis. Lei Liu, Ming Zhou, Akao Zhu, Xiangxing Kong, Jingjing Wu, and Yeting Hu provided technical support. Xiaoxu Ge wrote the manuscript. All authors read and approved the final version of the manuscript.

## Funding

This work was supported by the Key R&D Program of Zhejiang (2023C03049), the Fundamental Research Funds for the Central Universities (No. 226–2022‐00009, 226–2024‐00176), the Noncommunicable Chronic Diseases‐National Science and Technology Major Project (No. 2024ZD0520100), and the Zhejiang Provincial Natural Science Foundation of China (LMS25H160014).

## Ethics Statement

This study was reviewed and approved by the Institutional Review Board of the Second Affiliated Hospital of Zhejiang University School of Medicine (Approval No.: 2025–0826). All patients included in the study provided signed informed consent.

## Conflicts of Interest

The authors declare no conflicts of interest.

## Supporting information


**Figure S1:** Multivariate cox regression analysis for prognostic factors of progression free survival. (A) Forest plot of independent prognostic factors for progression free survival (PFS), red dots indicate unfavorable prognostic factors. (B) The Kaplan–Meier curve shows factors significantly affecting PFS in the multivariate Cox regression analysis. (C) Bar charts separately illustrate the median survival times of independent prognostic factors for PFS.

**Figure S2:** Predictive performance of nomogram models for PFS. (A) Visual representation of the nomogram for PFS prediction. (B) ROC curves and corresponding AUC values for PFS models at 1, 2, and 3 years. (C) Kaplan–Meier survival curves stratified by low‐risk and high‐risk groups based on the nomogram models. (D) Comparison of AUC values for the nomogram models versus other independent prognostic factors at 1, 2, and 3 years. (E) DCA curves illustrating the net benefit of nomogram models for PFS at varying threshold probabilities across 1–3 years. (F) Superiority of the nomogram model in predicting PFS compared to established scoring systems over 1–3 years.

**Figure S3:** Calibration curves for nomogram models across 1–3 years. (A, B) Calibration curves for OS and PFS, respectively, illustrating the model's predictive accuracy over time.

**Table S1:** Univariate and multivariate cox regression analysis for progression free survival outcomes.

**Table S2:** Subgroup analysis of the effect of HIPEC on OS in CRC‐SPM.

## Data Availability

All data generated or analyzed during this study is included in this published article.
