# Supplementary figures and images for: Prognostic Factors and Nomogram‐Based Prediction Models for Colorectal Cancer Patients With Synchronous Peritoneal Metastasis Undergoing Cytoreductive Surgery: A Retrospective Cohort Study
Source: Cancer Med. 2025 Dec 26;15(1):e71464. doi: 10.1002/cam4.71464 (PMC12742547; doi:10.1002/cam4.71464)

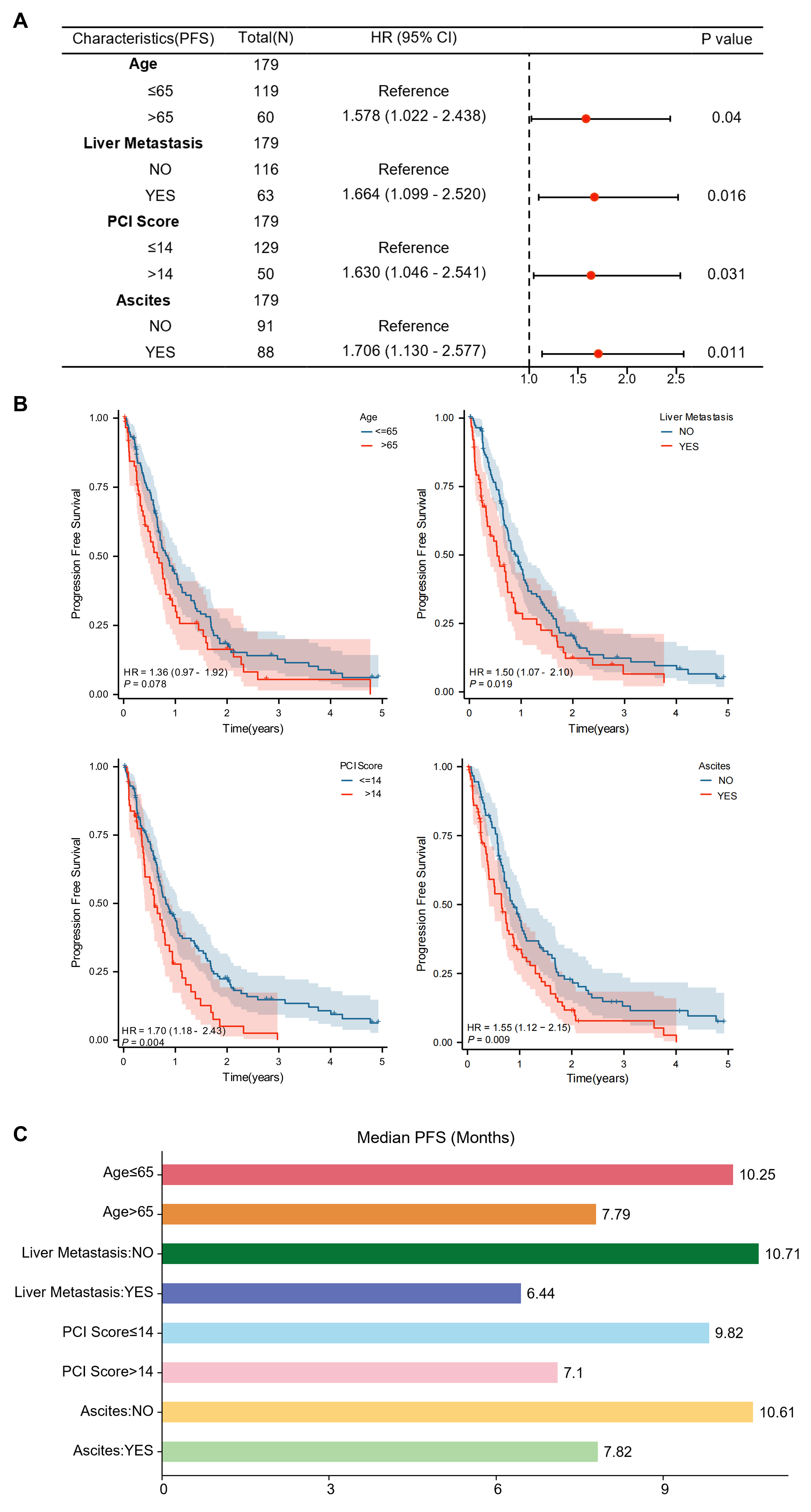

Supplement: Supplementary file 1 — Figure S1: Multivariate cox regression analysis for prognostic factors of progression free survival. (A) Forest plot of independent prognostic factors for progression free survival (PFS), red dots indicate unfavorable prognostic factors. (B) The Kaplan–Meier curve shows factors significantly affecting PFS in the multivariate Cox regression analysis. (C) Bar charts separately illustrate the median survival times of independent prognostic factors for PFS. [file CAM4-15-e71464-s002.tif]

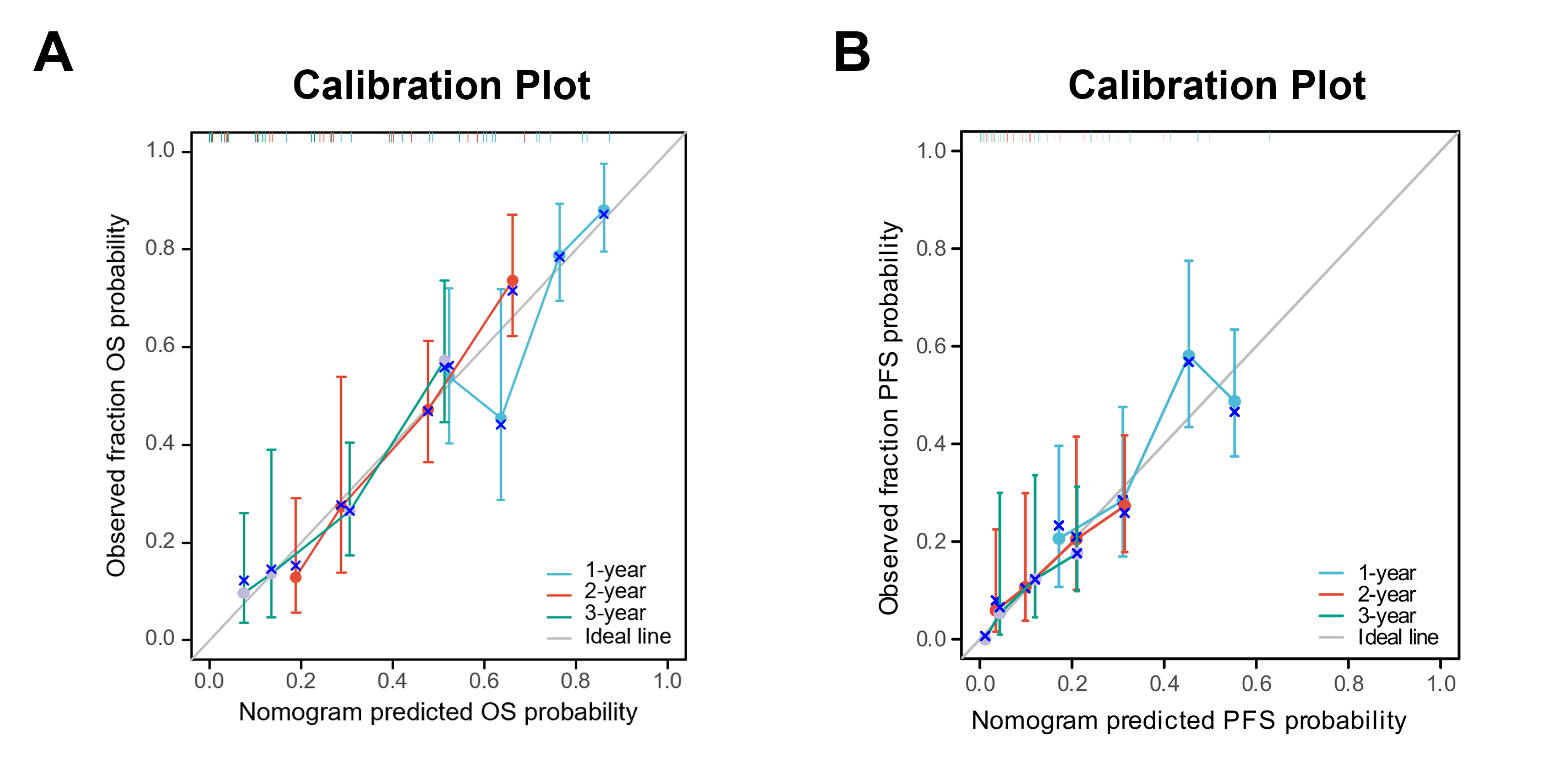

Supplement: Supplementary file 2 — Figure S2: Predictive performance of nomogram models for PFS. (A) Visual representation of the nomogram for PFS prediction. (B) ROC curves and corresponding AUC values for PFS models at 1, 2, and 3 years. (C) Kaplan–Meier survival curves stratified by low‐risk and high‐risk groups based on the nomogram models. (D) Comparison of AUC values for the nomogram models versus other independent prognostic factors at 1, 2, and 3 years. (E) DCA curves illustrating the net benefit of nomogram models for PFS at varying threshold probabilities across 1–3 years. (F) Superiority of the nomogram model in predicting PFS compared to established scoring systems over 1–3 years. [file CAM4-15-e71464-s004.tif]
