# Supplementary material for: Prognostic Factors and Nomogram‐Based Prediction Models for Colorectal Cancer Patients With Synchronous Peritoneal Metastasis Undergoing Cytoreductive Surgery: A Retrospective Cohort Study
Source: Cancer Med. 2025 Dec 26;15(1):e71464. doi: 10.1002/cam4.71464 (PMC12742547; doi:10.1002/cam4.71464)
Supplement: Supplementary file 3 — Figure S3: Calibration curves for nomogram models across 1–3 years. (A, B) Calibration curves for OS and PFS, respectively, illustrating the model's predictive accuracy over time. [file CAM4-15-e71464-s005.pdf]

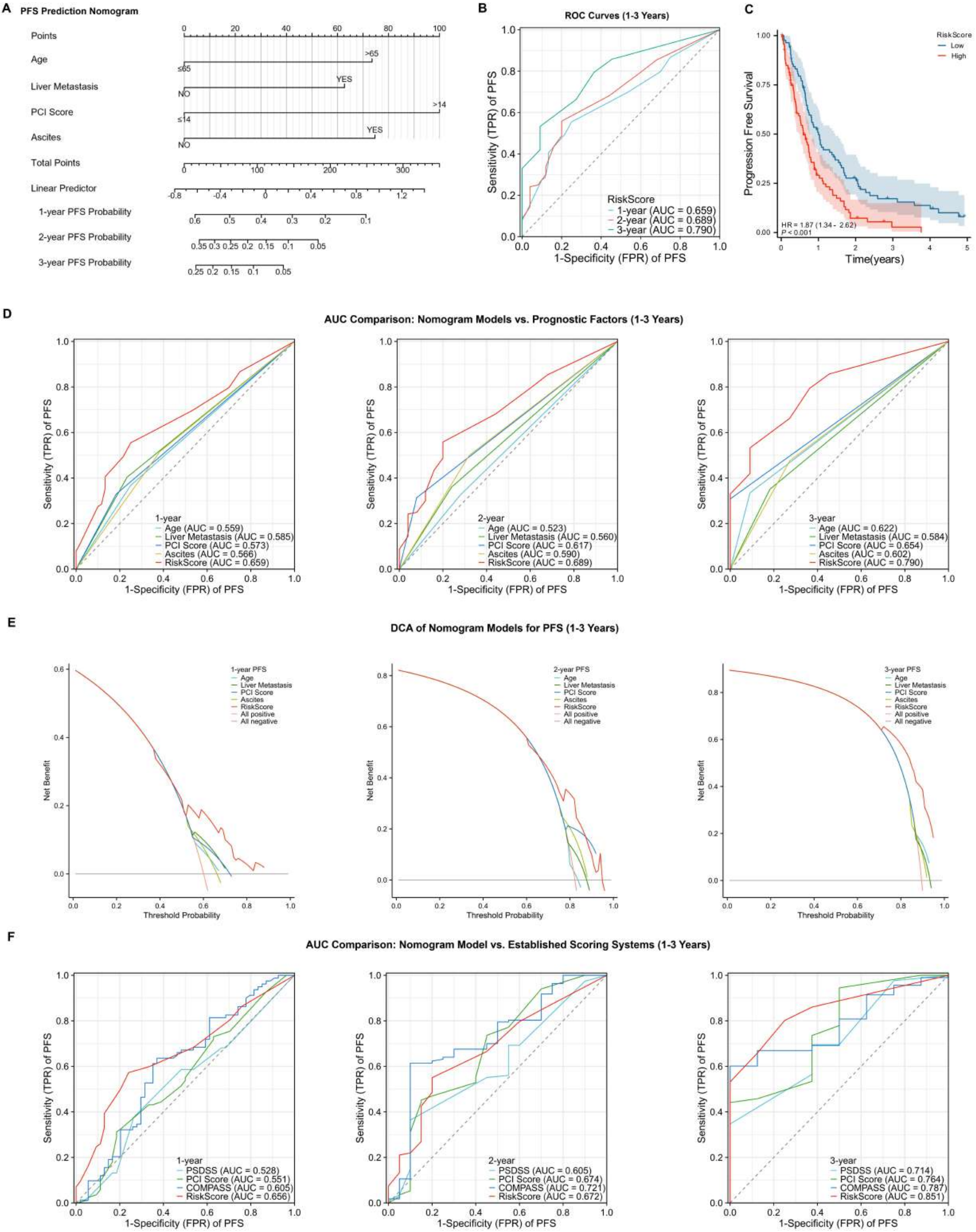
A
PFS Prediction Nomogram
Points
Age
>65
≤65
Liver Metastasis
YES
NO
PCI Score
>14
≤14
Ascites
YES
NO
Total Points
Linear Predictor
1-year PFS Probability
2-year PFS Probability
3-year PFS Probability
B
ROC Curves (1-3 Years)
Sensitivity (TPR) of PFS
1-Specificity (FPR) of PFS
RiskScore
1-year (AUC = 0.659)
2-year (AUC = 0.689)
3-year (AUC = 0.790)
C
RiskScore
Low
High
Progression Free Survival
HR = 1.87 (1.34 - 2.62)
P < 0.001
Time(years)
D
AUC Comparison: Nomogram Models vs. Prognostic Factors (1-3 Years)
1-year
Age (AUC = 0.559)
Liver Metastasis (AUC = 0.585)
PCI Score (AUC = 0.573)
Ascites (AUC = 0.566)
RiskScore (AUC = 0.659)
2-year
Age (AUC = 0.523)
Liver Metastasis (AUC = 0.560)
PCI Score (AUC = 0.617)
Ascites (AUC = 0.590)
RiskScore (AUC = 0.689)
3-year
Age (AUC = 0.622)
Liver Metastasis (AUC = 0.584)
PCI Score (AUC = 0.654)
Ascites (AUC = 0.602)
RiskScore (AUC = 0.790)
E
DCA of Nomogram Models for PFS (1-3 Years)
1-year PFS
2-year PFS
3-year PFS
Age
Liver Metastasis
PCI Score
Ascites
RiskScore
All positive
All negative
Net Benefit
Threshold Probability
F
AUC Comparison: Nomogram Model vs. Established Scoring Systems (1-3 Years)
1-year
PSDSS (AUC = 0.528)
PCI Score (AUC = 0.551)
COMPASS (AUC = 0.605)
RiskScore (AUC = 0.656)
2-year
PSDSS (AUC = 0.605)
PCI Score (AUC = 0.674)
COMPASS (AUC = 0.721)
RiskScore (AUC = 0.672)
3-year
PSDSS (AUC = 0.714)
PCI Score (AUC = 0.764)
COMPASS (AUC = 0.787)
RiskScore (AUC = 0.851)
