# Supplementary material for: Prognostic Factors and Nomogram‐Based Prediction Models for Colorectal Cancer Patients With Synchronous Peritoneal Metastasis Undergoing Cytoreductive Surgery: A Retrospective Cohort Study
Source: Cancer Med. 2025 Dec 26;15(1):e71464. doi: 10.1002/cam4.71464 (PMC12742547; doi:10.1002/cam4.71464)
Supplement: Supplementary file 4 — Table S1: Univariate and multivariate cox regression analysis for progression free survival outcomes. [file CAM4-15-e71464-s001.docx]

**Supplementary Table 1: Univariate and Multivariate Cox Regression Analysis for Progression Free Survival Outcomes**

| Characteristics | Total(N) | Univariate analysis | |  | Multivariate analysis | |
| --- | --- | --- | --- | --- | --- | --- |
|  |  | Hazard ratio (95% CI) | P value |  | Hazard ratio (95% CI) | P value |
| Year of CRS performed | 179 |  |  |  |  |  |
| 2010-2016 | 34 | Reference |  |  |  |  |
| 2017-2022 | 145 | 1.030 (0.684 - 1.549) | 0.889 |  |  |  |
| Gender | 179 |  |  |  |  |  |
| Female | 79 | Reference |  |  |  |  |
| Male | 100 | 0.880 (0.636 - 1.217) | 0.440 |  |  |  |
| Age | 179 |  |  |  |  |  |
| ≤65 | 119 | Reference |  |  | Reference |  |
| >65 | 60 | 1.363 (0.966 - 1.924) | **0.078** |  | 1.578 (1.022 - 2.438) | **0.040** |
| BMI | 177 |  |  |  |  |  |
| <24 | 124 | Reference |  |  |  |  |
| ≥24 | 53 | 0.819 (0.575 - 1.167) | 0.268 |  |  |  |
| Smoking History | 179 |  |  |  |  |  |
| NO | 126 | Reference |  |  |  |  |
| YES | 53 | 0.813 (0.567 - 1.166) | 0.261 |  |  |  |
| Alcohol History | 179 |  |  |  |  |  |
| NO | 132 | Reference |  |  |  |  |
| YES | 47 | 0.755 (0.518 - 1.100) | 0.143 |  |  |  |
| Hypertension History | 179 |  |  |  |  |  |
| NO | 129 | Reference |  |  |  |  |
| YES | 50 | 1.119 (0.783 - 1.600) | 0.538 |  |  |  |
| Diabetes History | 179 |  |  |  |  |  |
| NO | 158 | Reference |  |  | Reference |  |
| YES | 21 | 1.567 (0.964 - 2.549) | **0.070** |  | 1.362 (0.734 - 2.525) | 0.327 |
| Liver Metastasis | 179 |  |  |  |  |  |
| NO | 116 | Reference |  |  | Reference |  |
| YES | 63 | 1.498 (1.067 - 2.101) | **0.019** |  | 1.664 (1.099 - 2.520) | **0.016** |
| Preoperative CEA | 164 |  |  |  |  |  |
| ≤5 ng/mL | 55 | Reference |  |  |  |  |
| >5 ng/mL | 109 | 1.077 (0.754 - 1.539) | 0.684 |  |  |  |
| Preoperative CA199 | 162 |  |  |  |  |  |
| ≤37 U/mL | 80 | Reference |  |  |  |  |
| >37 U/mL | 82 | 0.893 (0.635 - 1.255) | 0.513 |  |  |  |
| Preoperative CA125 | 160 |  |  |  |  |  |
| ≤35 U/mL | 89 | Reference |  |  |  |  |
| >35 U/mL | 71 | 1.116 (0.790 - 1.576) | 0.534 |  |  |  |
| Preoperative CA242 | 154 |  |  |  |  |  |
| ≤20 U/mL | 82 | Reference |  |  |  |  |
| >20 U/mL | 72 | 1.019 (0.717 - 1.449) | 0.914 |  |  |  |
| Preoperative Chemotherapy | 179 |  |  |  |  |  |
| NO | 146 | Reference |  |  |  |  |
| YES | 33 | 1.058 (0.692 - 1.618) | 0.793 |  |  |  |
| Preoperative Radiotherapy | 179 |  |  |  |  |  |
| NO | 177 | Reference |  |  |  |  |
| YES | 2 | 1.292 (0.318 - 5.242) | 0.720 |  |  |  |
| Preoperative Targeted Therapy | 179 |  |  |  |  |  |
| NO | 161 | Reference |  |  |  |  |
| YES | 18 | 1.345 (0.787 - 2.299) | 0.278 |  |  |  |
| CRS Type | 179 |  |  |  |  |  |
| Open surgery | 113 | Reference |  |  |  |  |
| Laparoscopic surgery | 66 | 0.999 (0.714 - 1.399) | 0.996 |  |  |  |
| Acute Abdominal Symptoms | 179 |  |  |  |  |  |
| NO | 130 | Reference |  |  |  |  |
| YES | 49 | 0.726 (0.492 - 1.069) | 0.105 |  |  |  |
| BRAF Mutation Status | 145 |  |  |  |  |  |
| BRAF wild-type | 133 | Reference |  |  | Reference |  |
| BRAF V600E mutation | 12 | 1.834 (0.954 - 3.525) | **0.069** |  | 1.136 (0.551 - 2.343) | 0.729 |
| Mismatch Repair Gene Status | 163 |  |  |  |  |  |
| pMMR | 156 | Reference |  |  |  |  |
| dMMR | 7 | 1.277 (0.562 - 2.903) | 0.560 |  |  |  |
| T Stage | 178 |  |  |  |  |  |
| T2-T3 | 45 | Reference |  |  |  |  |
| T4 | 133 | 0.926 (0.640 - 1.340) | 0.683 |  |  |  |
| Tumor Margin Status of Primary Lesion | 178 |  |  |  |  |  |
| R0 | 174 | Reference |  |  |  |  |
| R1-R2 | 4 | 1.446 (0.457 - 4.574) | 0.530 |  |  |  |
| Tumor Size | 173 |  |  |  |  |  |
| ≤3.5cm | 50 | Reference |  |  | Reference |  |
| >3.5cm | 123 | 1.384 (0.963 - 1.989) | **0.079** |  | 1.174 (0.730 - 1.889) | 0.508 |
| Neural Invasion in Primary Tumor | 165 |  |  |  |  |  |
| NO | 51 | Reference |  |  |  |  |
| YES | 114 | 1.138 (0.789 - 1.641) | 0.488 |  |  |  |
| Vascular Invasion in Primary Tumor | 169 |  |  |  |  |  |
| NO | 65 | Reference |  |  | Reference |  |
| YES | 104 | 1.341 (0.952 - 1.889) | **0.094** |  | 1.068 (0.667 - 1.710) | 0.785 |
| N Stage | 175 |  |  |  |  |  |
| N0-N1 | 96 | Reference |  |  | Reference |  |
| N2 | 79 | 1.373 (0.981 - 1.920) | **0.064** |  | 1.301 (0.795 - 2.129) | 0.295 |
| Number of Lymph Nodes Resected | 174 |  |  |  |  |  |
| ≤13 | 63 | Reference |  |  | Reference |  |
| >13 | 111 | 0.732 (0.519 - 1.031) | **0.074** |  | 0.698 (0.449 - 1.084) | 0.109 |
| Location of Primary Tumor | 179 |  |  |  |  |  |
| Right-sided colon | 90 | Reference |  |  |  |  |
| Left-sided colon | 89 | 1.228 (0.888 - 1.700) | 0.215 |  |  |  |
| Pathological Type | 179 |  |  |  |  |  |
| Adenocarcinoma | 139 | Reference |  |  |  |  |
| Mucinous adenocarcinoma | 40 | 1.258 (0.861 - 1.838) | 0.235 |  |  |  |
| Pathological Differentiation Degree | 152 |  |  |  |  |  |
| Well - Moderately Differentiated | 96 | Reference |  |  |  |  |
| Poorly Differentiated | 56 | 1.136 (0.785 – 1.644) | 0.500 |  |  |  |
| Macroscopic Type | 159 |  |  |  |  |  |
| infiltrative | 1 | Reference |  |  |  |  |
| ulcerative | 118 | 1.882 (0.261 - 13.551) | 0.530 |  |  |  |
| protruding | 40 | 1.836 (0.250 - 13.467) | 0.550 |  |  |  |
| CC Score | 179 |  |  |  |  |  |
| CC0 | 144 | Reference |  |  |  |  |
| CC1-CC3 | 35 | 1.176 (0.779 - 1.775) | 0.440 |  |  |  |
| PCI Score | 179 |  |  |  |  |  |
| ≤14 | 129 | Reference |  |  | Reference |  |
| >14 | 50 | 1.697 (1.183 - 2.432) | **0.004** |  | 1.630 (1.046 - 2.541) | **0.031** |
| Ascites | 179 |  |  |  |  |  |
| NO | 91 | Reference |  |  | Reference |  |
| YES | 88 | 1.550 (1.117 - 2.152) | **0.009** |  | 1.706 (1.130 - 2.577) | **0.011** |
| Invasion of Small Intestine | 179 |  |  |  |  |  |
| NO | 145 | Reference |  |  |  |  |
| YES | 34 | 1.109 (0.742 - 1.657) | 0.614 |  |  |  |
| Intraoperative Blood Loss | 178 |  |  |  |  |  |
| <100mL | 76 | Reference |  |  |  |  |
| ≥100mL | 102 | 0.929 (0.667 - 1.292) | 0.661 |  |  |  |
| HIPEC Performed | 179 |  |  |  |  |  |
| NO | 115 | Reference |  |  |  |  |
| YES | 64 | 0.761 (0.543 - 1.066) | 0.112 |  |  |  |
| Postoperative Complications | 179 |  |  |  |  |  |
| NO | 165 | Reference |  |  |  |  |
| YES | 14 | 1.064 (0.520 - 2.175) | 0.865 |  |  |  |
| Average Postoperative Hospital Stay | 179 |  |  |  |  |  |
| <10 days | 96 | Reference |  |  |  |  |
| ≥10 days | 83 | 0.921 (0.665 - 1.274) | 0.617 |  |  |  |
| Postoperative Chemotherapy | 174 |  |  |  |  |  |
| NO | 18 | Reference |  |  |  |  |
| YES | 156 | 0.769 (0.425 - 1.392) | 0.386 |  |  |  |
| Postoperative Radiotherapy | 174 |  |  |  |  |  |
| NO | 164 | Reference |  |  |  |  |
| YES | 10 | 0.904 (0.460 - 1.780) | 0.771 |  |  |  |
| Postoperative Immunotherapy | 173 |  |  |  |  |  |
| NO | 154 | Reference |  |  |  |  |
| YES | 19 | 0.798 (0.486 - 1.312) | 0.374 |  |  |  |
| Postoperative Targeted Therapy | 173 |  |  |  |  |  |
| NO | 58 | Reference |  |  |  |  |
| YES | 115 | 0.997 (0.691 - 1.439) | 0.987 |  |  |  |

^†^Mucinous adenocarcinoma category includes mucinous adenocarcinoma and signet ring cell carcinoma in the dichotomous classification.
