# Supplementary material for: Prognostic Factors and Nomogram‐Based Prediction Models for Colorectal Cancer Patients With Synchronous Peritoneal Metastasis Undergoing Cytoreductive Surgery: A Retrospective Cohort Study
Source: Cancer Med. 2025 Dec 26;15(1):e71464. doi: 10.1002/cam4.71464 (PMC12742547; doi:10.1002/cam4.71464)
Supplement: Supplementary file 5 — Table S2: Subgroup analysis of the effect of HIPEC on OS in CRC‐SPM. [file CAM4-15-e71464-s003.docx]

**Supplementary Table 2: Subgroup Analysis of the Effect of HIPEC on OS in CRC-SPM**

| Characteristics | Total(N) | CRS | CRS+HIPEC | Hazard ratio (95% CI) | | P for interaction |
| --- | --- | --- | --- | --- | --- | --- |
| Total patients number (n=218) | 218 | 116/145 | 52/73 | | 0.54 (0.39-0.76) |  |
| Year of CRS performed | 218 |  |  | |  | 0.710 |
| 2010-2016 | 48 | 27/29 | 16/19 | | 0.48 (0.26-0.91) |  |
| 2017-2022 | 170 | 89/116 | 36/54 | | 0.56 (0.38-0.83) |  |
| Gender | 218 |  |  | |  | 0.621 |
| Female | 99 | 47/60 | 30/39 | | 0.58 (0.36-0.93) |  |
| Male | 119 | 69/85 | 22/34 | | 0.49 (0.30-0.80) |  |
| Age | 218 |  |  | |  | 0.079 |
| ≤65 | 136 | 59/84 | 36/52 | | 0.95 (0.45-2.02) |  |
| >65 | 82 | 57/61 | 16/21 | | 0.22 (0.05-0.94) |  |
| BMI | 214 |  |  | |  | 0.447 |
| <24 | 152 | 85/105 | 35/47 | | 0.50 (0.33-0.74) |  |
| ≥24 | 62 | 28/36 | 17/26 | | 0.66 (0.35-1.21) |  |
| Smoking History | 218 |  |  | |  | 0.670 |
| NO | 158 | 77/98 | 44/60 | | 0.54 (0.37-0.80) |  |
| YES | 60 | 39/47 | 8/13 | | 0.46 (0.21-0.99) |  |
| Alcohol History | 218 |  |  | |  | 0.479 |
| NO | 164 | 85/106 | 41/58 | | 0.49 (0.33-0.72) |  |
| YES | 54 | 31/39 | 11/15 | | 0.66 (0.33-1.31) |  |
| Hypertension History | 218 |  |  | |  | 0.887 |
| NO | 156 | 75/100 | 39/56 | | 0.56 (0.37-0.83) |  |
| YES | 62 | 41/45 | 13/17 | | 0.56 (0.30-1.05) |  |
| Diabetes History | 218 |  |  | |  | 0.546 |
| NO | 187 | 94/121 | 46/66 | | 0.54 (0.38-0.78) |  |
| YES | 31 | 22/24 | 6/7 | | 0.70 (0.28-1.74) |  |
| Liver Metastasis | 218 |  |  | |  | 0.081 |
| NO | 136 | 54/72 | 48/64 | | 0.69 (0.46-1.02) |  |
| YES | 82 | 62/73 | 4/9 | | 0.31 (0.11-0.85) |  |
| Preoperative CEA | 197 |  |  | |  | 0.735 |
| ≤5 ng/mL | 62 | 29/38 | 18/24 | | 0.60 (0.33-1.10) |  |
| >5 ng/mL | 135 | 75/93 | 30/42 | | 0.54 (0.35-0.83) |  |
| Preoperative CA199 | 194 |  |  | |  | 0.503 |
| ≤37 U/mL | 92 | 44/57 | 25/35 | | 0.62 (0.38-1.02) |  |
| >37 U/mL | 102 | 58/72 | 23/30 | | 0.55 (0.33-0.91) |  |
| Preoperative CA125 | 192 |  |  | |  | 0.644 |
| ≤35 U/mL | 105 | 59/71 | 22/34 | | 0.45 (0.27-0.73) |  |
| >35 U/mL | 87 | 44/58 | 24/29 | | 0.63 (0.38-1.06) |  |
| Preoperative CA242 | 186 |  |  | |  | 0.816 |
| ≤20 U/mL | 95 | 44/56 | 28/39 | | 0.56 (0.34-0.91) |  |
| >20 U/mL | 91 | 55/69 | 17/22 | | 0.60 (0.35-1.05) |  |
| Preoperative Chemotherapy | 218 |  |  | |  | 0.774 |
| NO | 178 | 94/118 | 44/60 | | 0.52 (0.36-0.75) |  |
| YES | 40 | 22/27 | 8/13 | | 0.61 (0.27-1.36) |  |
| Preoperative Radiotherapy | 218 |  |  | |  |  |
| NO | 216 | 114/143 | 52/73 | | 0.54 (0.39-0.76) | NA |
| YES | 2 | 2/2 | 0/0 | |  |  |
| Preoperative Targeted Therapy | 218 |  |  | |  | 0.717 |
| NO | 195 | 102/130 | 47/65 | | 0.56 (0.39-0.80) |  |
| YES | 23 | 14/15 | 5/8 | | 0.45 (0.16-1.26) |  |
| CRS Type | 218 |  |  | |  | 0.728 |
| Open surgery | 141 | 74/92 | 36/49 | | 0.59 (0.39-0.88) |  |
| Laparoscopic surgery | 77 | 42/53 | 16/24 | | 0.44 (0.24-0.80) |  |
| Acute Abdominal Symptoms | 218 |  |  | |  | 0.428 |
| NO | 153 | 77/97 | 41/56 | | 0.58 (0.39-0.85) |  |
| YES | 65 | 39/48 | 11/17 | | 0.44 (0.22-0.88) |  |
| BRAF Mutation Status | 179 |  |  | |  | 0.539 |
| BRAF wild-type | 166 | 86/107 | 44/59 | | 0.60 (0.42-0.87) |  |
| BRAF V600E mutation | 13 | 9/11 | 2/2 | | 1.16 (0.24-5.56) |  |
| Mismatch Repair Gene Status | 191 |  |  | |  | **0.042** |
| pMMR | 184 | 92/119 | 47/65 | | 0.55 (0.38-0.78) |  |
| dMMR | 7 | 4/4 | 1/3 | | 0.27 (0.03-2.52) |  |
| T Stage | 215 |  |  | |  | 0.322 |
| T2-T3 | 51 | 27/32 | 12/19 | | 0.47 (0.23-0.96) |  |
| T4 | 164 | 88/112 | 39/52 | | 0.60 (0.41-0.89) |  |
| Tumor Margin Status of Primary Lesion | 214 |  |  | |  | 0.929 |
| R0 | 207 | 111/139 | 49/68 | | 0.53 (0.38-0.75) |  |
| R1-R2 | 7 | 4/4 | 3/3 | | 0.97 (0.17-5.48) |  |
| Tumor Size | 206 |  |  | |  | **0.033** |
| ≤3.5cm | 57 | 21/32 | 18/25 | | 0.96 (0.51-1.81) |  |
| >3.5cm | 149 | 90/106 | 32/43 | | 0.45 (0.30-0.68) |  |
| Neural Invasion in Primary Tumor | 193 |  |  | |  | 0.553 |
| NO | 59 | 30/38 | 16/21 | | 0.70 (0.38-1.29) |  |
| YES | 134 | 73/92 | 31/42 | | 0.55 (0.36-0.85) |  |
| Vascular Invasion in Primary Tumor | 199 |  |  | |  | 0.384 |
| NO | 77 | 36/48 | 17/29 | | 0.46 (0.26-0.83) |  |
| YES | 122 | 69/85 | 32/37 | | 0.74 (0.49-1.14) |  |
| N Stage | 210 |  |  | |  | 0.546 |
| N0-N1 | 120 | 61/81 | 26/39 | | 0.52 (0.32-0.83) |  |
| N2 | 90 | 53/62 | 23/28 | | 0.65 (0.40-1.06) |  |
| Number of Lymph Nodes Resected | 208 |  |  | |  | 0.669 |
| ≤13 | 83 | 50/58 | 21/25 | | 0.56 (0.33-0.95) |  |
| >13 | 125 | 64/84 | 27/41 | | 0.52 (0.33-0.82) |  |
| Location of Primary Tumor | 218 |  |  | |  | 0.144 |
| Right-sided colon | 111 | 53/70 | 32/41 | | 0.70 (0.45-1.10) |  |
| Left-sided colon | 107 | 63/75 | 20/32 | | 0.37 (0.22-0.63) |  |
| Pathological Type |  |  |  | |  | 0.953 |
| Adenocarcinoma | 166 | 95/121 | 31/45 | | 0.52 (0.34-0.78) |  |
| Mucinous adenocarcinoma | 52 | 21/24 | 21/28 | | 0.46 (0.24-0.87) |  |
| Pathological Differentiation Degree | 180 |  |  | |  | 0.247 |
| Well- Moderately Differentiated | 116 | 72/89 | 16/27 | | 0.36 (0.20-0.63) |  |
| Poorly Differentiated | 64 | 33/44 | 16/20 | | 0.63 (0.34-1.15) |  |
| Macroscopic Type | 188 |  |  | |  | 0.777 |
| infiltrative | 1 | 0/0 | 1/1 | |  |  |
| ulcerative | 136 | 75/95 | 30/41 | | 0.61 (0.39-0.93) |  |
| protruding | 51 | 30/36 | 11/15 | | 0.48 (0.23-1.03) |  |
| CC Score | 218 |  |  | |  | 0.347 |
| CC0 | 172 | 89/115 | 38/57 | | 0.50 (0.34-0.73) |  |
| CC1-CC3 | 46 | 27/30 | 14/16 | | 0.70 (0.36-1.33) |  |
| PCI Score | 218 |  |  | |  | 0.055 |
| ≤14 | 153 | 85/111 | 23/42 | | 0.37 (0.23-0.59) |  |
| >14 | 65 | 31/34 | 29/31 | | 0.71 (0.43-1.19) |  |
| Ascites | 218 |  |  | |  | 0.844 |
| NO | 105 | 52/66 | 26/39 | | 0.52 (0.32-0.84) |  |
| YES | 113 | 64/79 | 26/34 | | 0.62 (0.39-0.98) |  |
| Invasion of Small Intestine | 218 |  |  | |  | 0.075 |
| NO | 170 | 86/109 | 42/61 | | 0.47 (0.32-0.69) |  |
| YES | 48 | 30/36 | 10/12 | | 0.99 (0.48-2.03) |  |
| Intraoperative Blood Loss | 216 |  |  | |  | 0.355 |
| <100mL | 89 | 47/65 | 19/24 | | 0.66 (0.38-1.13) |  |
| ≥100mL | 127 | 68/78 | 33/49 | | 0.47 (0.31-0.72) |  |
